# Enhanced Functionality and Bio-Accessibility of Composite Pomegranate Peel Extract-Enriched “Boba Balls”

**DOI:** 10.3390/foods11233785

**Published:** 2022-11-24

**Authors:** Ayse Neslihan Dundar, Kubra Uzuner, Mahmud Ekrem Parlak, Oya Irmak Sahin, Furkan Turker Saricaoglu, Senay Simsek

**Affiliations:** 1 Department of Food Engineering, Faculty of Engineering and Natural Science, Bursa Technical University, 16310 Bursa, Turkey; 2 Department of Chemical Engineering, Faculty of Engineering, Yalova University, 77200 Yalova, Turkey; 3 Whistler Center for Carbohydrate Research, Department of Food Science, Purdue University, West Lafayette, IN 47907, USA

**Keywords:** Boba, alginate beads, pomegranate peel extract, in vitro digestion, release kinetic

## Abstract

“Boba balls” or pearls have recently gained popularity for beverages or food toppings. “Boba balls” could be developed into functional foods by the encapsulation of bioactive compounds. In this study, gelatin/sodium alginate composite “Boba balls” enriched with pomegranate peel extract (PPE) at different concentrations (0, 1, 2, and 3%) were prepared. They were characterized in terms of physical, rheological, textural, morphological, and sensory properties, as well as in vitro digestion, bio-accessibility, and release kinetic of PPE. Adding PPE improved the “Boba” mix’s viscoelasticity and decreased the “Boba balls”’ hardness. The increasing PPE ratio significantly (*p* < 0.05) increased the antioxidant capacity and total phenolic content. The addition of PPE preserved the spherical shape of the “Boba balls”, and as the PPE ratio increased, new junction zones were observed in SEM images. The in vitro digestibility of PPE was significantly (*p* < 0.05) improved by preserving PPE from the mouth and gastric medium, and “Boba balls” showed the highest release and bio-accessibility in the intestinal medium. Consequently, PPE as a by-product could be successfully used at 2% concentration for enhancing the functionality and bio-accessibility of “Boba balls” without affecting sensory properties.

## 1. Introduction

Traditional bubble tea, also known as Boba tea, is a drink made from tea, milk, syrup, and tapioca pearls. The traditional “Boba balls” are made from gelatinized tapioca starch that has been formed into a pearl shape. Customarily, they acquire their color and flavor from brown sugar, but may incorporate other colors or flavors [1]. “Boba balls” can also be produced with various carbohydrate and/or protein-based gel-forming polymers that can complex with sodium alginate and are generally known for high elasticity, resilient mouthfeel, edible convenience, and low calorie content [2]. “Boba balls” began to become widespread in Taiwan in the early 1980s and are usually consumed with milk tea. Later, they spread worldwide, especially in America and Europe, and the market value in 2019 reached $2.09 billion. It is predicted that the estimated market value will reach $3.29 billion in 2027 [1]. “Boba balls” are also called “pearls” because they are structurally bead-shaped and are consumed by mixing with hot or cold tea, milk tea, and fruit juices.

While there are many studies on alginate encapsulation by calcium alginate gels [3,4,5] there is much less information about alginate gel-based “Boba balls”. Alginate gel-based “Boba balls” have been investigated recently to determine optimum technological parameters and for the inclusion of various flavor, aroma, color, or nutritive components [6]. Additionally, shelf-life studies have shown that the sensory quality of “Boba balls” decreases over time and storage conditions impact the degradation of sensory values [6].

The spherical balls can be produced with fruits providing different aromas; these structures can also be produced by using herbal extracts with functional properties, such as phenolic compounds, antioxidants, anthocyanins, etc. Considering the consumption and market value of “Boba balls”, it is obvious that enriching these products with plant extracts rich in functional properties will significantly contribute to human health. In a study, Liu, Xiao, Feng, Zhang, Li, Tu and Niu [2] produced “Boba balls” with konjac glucomannan/sodium alginate composite gels and enriched “Boba balls” with purple-potato-anthocyanin extracts to increase gastrointestinal digestion stability. The enrichment of “Boba balls” with herbal extracts obtained from food processing by-products will improve the nutritional properties of “Boba balls” as well as improve the bio-availability of herbal extracts.

Pomegranate (*Punica granatum* L.) peel is a standard by-product from the production of pomegranate juice, and the peel makes up 40–50% of the total weight of the pomegranate. There are numerous studies about the composition of pomegranate peel extract (PPE), and it was previously reported that PPE is rich in bioactive compounds, for example polyphenols, flavonoids, anthocyanin and tannins such as ellagic acid, tannins, gallic acid, and punicalagin A and punicalagin B [7]. Moreover, the antioxidant, antimicrobial, antiviral, antifungal, and anti-diabetic properties of PPE are highly related with these bioactive compounds. These functional and bioactive properties of PPE have increased the possibilities of incorporating PPE as an additive and/or supplement for the development of various functional foods [8].

The reason for choosing PPE in this study is that pomegranate peels, which are an important by-product of the fruit juice industry, have a very rich polyphenolic and tannin content when compared to other vegetables and fruits by-products. Additionally, the composition of the PPE and its effects on health have been clarified by many studies [7,8]. However, polyphenols and other bioactive compounds in PPE are highly sensitive to light, oxygen, pH, heat, pressure, etc., and encapsulation is a promising technique to protect these components not only from such environmental conditions but also from gastrointestinal digestion conditions.

The production of “Boba balls” is mainly similar to encapsulation methods since the core material, e.g., PPE, is covered by sodium alginate beads after being dropped into a CaCl_2_ solution. The reaction occurring between the sodium alginate and the CaCl_2_ is called ionic gelation. Thus, different core materials such as purple-sweet-potato anthocyanin [2] and orange flavor [6] have been encapsulated in the “Boba balls”. The encapsulation of active ingredients from food industry by-products in “Boba balls” will increase the utilization rate of active ingredients during the consumption of these products, thus enabling the consumption of healthier and bio-available products.

There is a growing global trend to reduce waste streams from the food industry by producing new value-added products. From this perspective, PPE is an important value-added product from the waste stream of pomegranate juice production as it is a good source of polyphenolics, antioxidants, phenolic acids, and tannins, etc. According to the literature, PPE is widely used for enhancing the shelf life of meat, chicken, fishery, dairy, and bakery products, as well as enhancing the antimicrobial properties of edible packaging materials [8]. However, there are no or very limited current literature studies about the production of “Boba balls” with PPE in order to increase the controlled release, in vitro digestibility, and bio-accessibility of PPE. As these parameters are a high priority for the development of functional foods, such as those proposed here, it will be essential to understand how the encapsulation of the PPE during the production of “Boba balls” impacts functionality. The aim of this work was to examine the antioxidant, phenolic content, bio-accessibility, and release kinetics of the “Boba balls” enriched with PPE at different ratios, as well as their rheological, textural, and morphological characterizations.

## 2. Materials and Methods

### 2.1. Materials

Sodium alginate (SA) (CAS number:9005-38-3), ethanol (absolute), Folin- Ciocalteu, 2,2’-azino-bis 3-ethylbenzothiazoline-6-sulphonic acid (ABTS), 2,2-diphenyl-1-picrylhydrazyl (DPPH), Neocuproine Cupric Ion Reducing Antioxidant Capacity (CUPRAC), and chemicals for simulating the in vitro digestion were acquired from Sigma-Aldrich (Darmstadt, Germany). Bovine gelatin at ~225 bloom and pomegranate (*Punica granatum* L.) peels were kindly provided by Bursa Gelatin (Bursa, Turkey) and Dohler (Denizli, Turkey), respectively. The pomegranate juice (PJ) was purchased from a local market in Bursa, Turkey, and it was used during the production of “Boba balls” for providing a pomegranate aroma. The peels were dried at 40 °C for 48 h in an air-circulated oven (Nukleon NST-120, Ankara, Turkey), and then dried peels were pulverized with a blender (Waring Commercial, PA, USA). The powdered pomegranate peels were filled in a Ziploc bags and stored at −18 °C.

### 2.2. Preparation of Pomegranate Peel Extract (PPE)

The preparation of pomegranate peel extract (PPE) was carried out following procedures from our previous study [9]. Briefly, a solution of ethanol/water (70/30; *v*/*v*) was mixed with powdered peels at 1/10 (*w*/*v*) ratio, and then homogenized (Velp Scientific, OV-5, Usmate, Italy) for 5 min at 10,000 rpm. In order to increase the extraction yield, the powdered peels underwent a further 15 min of ultrasonic homogenization (JP Selecta, CY-500, Barcelona, Spain) at 50% amplitude using a 7 mm titanium probe. An ice-water bath was used to maintain a temperature below 25 °C throughout the ultrasonic homogenization. Solid particles were removed by the centrifugation (Hettich, Universal 320, Kirchlengern, Germany) for 10 min at 9056× *g*, and the supernatant was evaporated at 45 °C using a vacuum rotary evaporator (Buchi, R-100, Lucerne, Switzerland). The evaporated extract was stored at −20 °C for 2 days before the freeze dried (Teknosem, TRS-2, Istanbul, Turkey) at −50 °C for 72 h and the resulting powders were stored at −18 °C in Ziploc bags.

### 2.3. Production of “Boba Balls”

**Figure 1:**
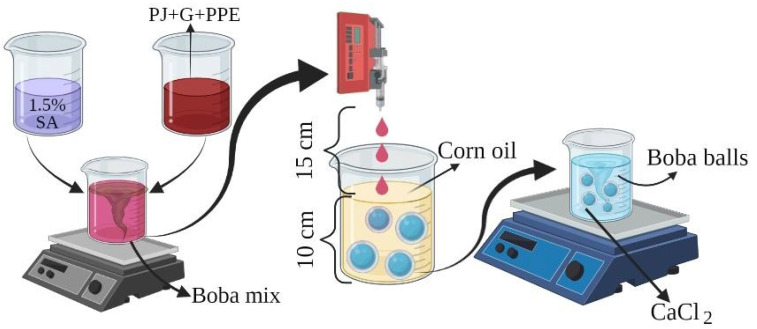
The schematic illustration of “Boba balls” production enriched with pomegranate peel extract (PPE).

The “Boba balls” production was performed by the methodology of Liu, Xiao, Feng, Zhang, Li, Tu and Niu [2], with the formulation and “Boba ball” production modifications illustrated in Figure 1. Firstly, 10 g of gelatin and PPE at 0, 1, 2 and 3% (*w*/*v*) concentrations were dissolved in 70 mL of pomegranate juice (PJ) for 5 min at 40 °C on a magnetic stirrer, and separately, SA (1.5 g) was dissolved in distilled water (30 mL) for 5 min at 70 °C on a magnetic stirrer. After cooling the SA solution at 40 °C, gelatin solution was combined with the SA solution before mixing for an additional 15 min at 40 °C. Then, the solution was cooled down to 20 °C and immediately transferred to a 20 mL syringe. The syringe was settled on the syringe pump (NE-400, Farmingdale, NY, USA), and the solution was fed drop by drop from a 15 cm distance into a 10 cm high bath of cooled corn oil at −9 °C. The gel “Boba balls” were collected with a stainless-steel filter spoon and mixed with 10% CaCl_2_ solution on a magnetic stirrer for 2 min. Finally, the “Boba balls” were collected by the filter spoon and washed twice with distilled water. The “Boba balls” were coded as C (control, without PPE), PPE1 (containing 1% PPE), PPE2 (containing 2% PPE), and PPE3 (containing 3% PPE), and samples of “Boba balls” were stored under refrigeration at 4 °C until further analysis. Prior to FT-IR and scanning electron microscopy, the “Boba balls” were stored at −20 °C for 2 days before the lyophilization at −50 °C for 72 h.

### 2.4. Rheological Characterization of “Boba” Mix

The gelation properties of “Boba” mix were evaluated with a controlled stress rheometer (Anton Paar, MCR302, Graz, Austria). A cone-plate geometry (25 mm diameter and 2° cone angle) was utilized for rheological evaluation. The composite solutions were held at 20 °C for 120 s on the flat surface for temperature equilibrium. The storage $\left(G^{\prime }\right)$ modulus and loss $\left(G^{\prime \prime }\right)$ modulus determined during the heating process from 20 to 50 °C and cooling process from 50 to 0 °C were measured at a heating ramp rate of 2 °C/min. Before the measurements, the linear viscoelastic range (LVR) was determined as 1% strain at 1 Hz frequency (data now shown).

### 2.5. Characterization of “Boba Balls”

#### 2.5.1. Total Phenolic Content (TPC), Antioxidant Capacity, and Anthocyanin Pigment Content

The TPC and antioxidant capacity of the PPE and “Boba balls” samples were measured. For this purpose, samples (1 g) were vortexed with ethanol/water (10 mL, 70/30, *v*/*v*) in a falcon tube, and then the mixture was kept in a shaking water bath at 25 °C for 2 h. After that, solids were separated from the supernatants of the samples by centrifugation for 10 min at 9000× *g*. Then the supernatant was used for the TPC and antioxidant capacity analysis. Analysis of TPC was performed based on the Folin–Ciocalteu method. The calculation of TPC was performed using gallic acid to construct the calibration curve [10], and the results were specified as mg gallic acid equivalent/g of dry matter.

The antioxidant capacity of PPE and “Boba balls” was determined with three different methods. Radical cation decolorization assay (2,2′-azino-bis 3-ethylbenzothiazoline-6-sulphonic acid), ABTS) [11], free radical scavenging assay (2,2-diphenyl-1-picrylhydrazyl, DPPH) [12] and cupric ion-reducing antioxidant capacity (CUPRAC) [13] were conducted on the PPE and “Boba balls”. The calibration curves were prepared using Trolox (6-hydroxy-2,5,7,8-tetramethylchromane-2-carboxylic acid), and the results were presented as µmole TE/g dry weight.

The total monomeric anthocyanin (TMA) content of “Boba balls” was established following the method and calculation of Lee, et al. [14], and results were stated as mg cyaniding-3-glucoside equivalents/g.

#### 2.5.2. Encapsulation Efficiency (EE) of Pomegranate Peel Extract in “Boba Balls”

The measurement of the encapsulation efficiency (EE) of PPE in “Boba balls” was determined based on the TPC of samples before and after the formation of “Boba balls”, and was calculated by the following formula:

(1) $$\begin{array}{c} EE\;\left(\%\right)\\ =\frac{Total\;phenolic\;contet\;of\;“Boba\;balls”}{Total\;phenolic\;content\;of\;“\mathrm{Boba}”\;\mathrm{mix}\;\mathrm{before}\;\mathrm{dropping}\;\mathrm{the}\;\mathrm{oil}}\\ \times 100 \end{array}$$

#### 2.5.3. Visual Appearance and Color

“Boba balls” were visually observed with a stereo zoom microscope (Leica EZ4E, Heerbrugg, Switzerland) at 8× magnification, and the images were captured with an integrated 5-megapixel camera. The color (L*, a*, and b*) values of “Boba balls” was assessed via a color spectrophotometer (X-Rite, Ci7800, Grand Rapids, MI, USA). For measurements, ten different “Boba balls” were used, and they were directly placed on the measuring cell, and the color values were recorded. Prior to the color measurement of the samples, calibration of the instrument was performed using a white standard plate (L* = 96.11, a* = −0.25, b* = 1.06). The total color difference (Δ*E*) of “Boba balls” based on the control sample was calculated according to following formula:

(2) $$∆E=\sqrt[{2}]{{\left(∆L^{*}\right)}^{2}+{\left(∆a^{*}\right)}^{2}+{\left(∆b^{*}\right)}^{2}}$$

#### 2.5.4. Texture Analysis

The texture profile analysis (TPA) of “Boba balls” was measured with a texture analyzer (Stable Micro Systems, TA-HD Plus, Godalming, UK) outfitted with a 36 mm diameter P/36R probe. “Boba balls” containing PPE extract were directly placed on the center of heavy-duty platform, and the probe was compressed to 50% of samples at a 5 mm/s test speed. The waiting time between the two cycles of the compression was 5 s, and 10 different “Boba balls” were subjected to testing at room temperature [2].

#### 2.5.5. FT-IR Spectrum

Lyophilized “Boba balls” and PPE extracts were converted into powder form with the help of a mortar, powdered samples (~20 mg) were directly placed in the sample chamber of the FT-IR device (TA, Nicolet iS50, San Marcos, CA, USA), and FT-IR spectra were taken directly with the help of an ATR diamond crystal at a 2 cm^−1^ scan resolution between 4000 and 400 cm^−1^ wavenumber range.

#### 2.5.6. Microstructure Analysis

The scanning electron microscope (SEM) (Carl Zeis, Gemini 300, Jena, Germany) was used for the visualization of the microstructure of “Boba balls”. “Boba balls” that broke after being immersed in liquid nitrogen for 1 min were fixed on the aluminum stubs by double-sided tape, followed by gold-palladium coating of samples in a low vacuum chamber (Leica, EM ACE200, Heerbrugg, Germany). Images were captured at an accelerating voltage of 20 kV. The surface microstructural images were captured at 100× and 500× magnifications.

#### 2.5.7. In Vitro Gastrointestinal Digestion

The in vitro gastrointestinal digestion of PPE and “Boba balls” was determined with the TPC and antioxidant capacity analyses (Section 2.5.1) after the digestion of samples in simulated gastrointestinal fluids. The salivary (SSF), gastric (SGF) and intestinal (SIF) fluids were formulated according to Minekus, et al. [15]. Firstly, 1 g samples were mixed with SSF and kept at 37 °C for 2 min in a water bath. Then, the solutions were transferred into SGF at pH 3, before being held at 100 rpm for 2 hours at 37 °C in a shaking water bath. Following this period, the solutions (10 mL) were transferred into the SIF and held at 100 rpm for 2 h at 37 °C in a shaking water bath. At the completion of the hydrolysis procedures, samples were centrifuged at 4 °C for 30 min at 9056× *g*, and then supernatants were stored at −18 °C. The bio-accessibility (%) was calculated by dividing the results obtained from the bio-accessible fraction by the sum of the extractable fraction and multiplying by 100.

#### 2.5.8. In Vitro Release Kinetic of “Boba Balls”

For the release kinetic study, the “Boba balls” in the gastric condition were sampled every 15 min for 120 min, and then every 30 min until 240 min. For the intestinal condition, samples were held in the gastric condition for 120 min and the sampling procedure was repeated as above. The release kinetics of phenolic compounds from “Boba balls” in the stomach and intestine mediums were evaluated by the Korsmeyer–Peppas (Equation (3)) and Peppas–Sahlin (Equation (4)) kinetic models, which are widely used in release kinetic studies, as well as the Makoid–Banakar model (Equation (5)):

(3) $$\frac{M_{t}}{M_{\infty }}=kt^{n}$$

(4) $$\frac{M_{t}}{M_{\infty }}=k_{1}t^{m}+k_{2}t^{2m}$$

(5) $$M_{t}=K_{MB}\times t^{n}\times e^{-ct}$$

where $M_{t}$ and $M_{\infty }$ refer to the releasing of TPC at *t* and infinite times, respectively. *k* and *n* refer to the kinetic constant depending on the geometrical and structural properties of particles, and release exponent, which shows the release mechanism, respectively. $k_{1}$, $k_{2}$, *m* and $K_{MB}$ are kinetic constants, diffusional exponent for Peppas–Sahlin model, and the Makoid–Banakar coefficient, respectively.

#### 2.5.9. Sensory Evaluation

The sensory panel was selected from the students and academicians of the Food Engineering Department at Bursa Technical University, Turkey. Panelists consisted of 15 women and 15 men between the ages of 19 and 42 years old that were semi-trained about the product and evaluation criteria before starting the evaluation. The products were evaluated by a five-point hedonic scale, where 1 = dislike very much, 2 = dislike slightly, 3 = neither like nor dislike, 4 = like slightly, and 5 = like very much. The evaluation was based on appearance, elasticity, taste, and aroma, general acceptability, and affordability. The samples were randomly numbered with three digits before serving to panelists. After the evaluation of each sample, the panelists rinsed their mouths with water.

### 2.6. Statistical Analysis

The production of “Boba balls” was performed in triplicate, and rheology, TPC, antioxidant capacity, EE, in vitro release, and sensory tests were conducted at least in triplicate, while texture and color analysis were measured for 10 samples per batch of “Boba balls”. The results of these tests are presented as mean ± standard deviation. A one-way analysis of variance was performed to determine any significant differences between treatments, followed by Duncan’s multiple range test at a 95% confidence interval with SPSS v21 (IBM, New York, NY, USA). OriginPro v8.5 (OriginLab Corp., Northampton, MA, USA) was used for the graphical visualization of the results. The release kinetic mathematical models were applied to experimental data by using MS Excel (v2016, Redmond, WA, USA) with DDSolver.

## 3. Results and Discussion

### 3.1. Rheological Properties of “Boba” Mix

**Figure 2:**
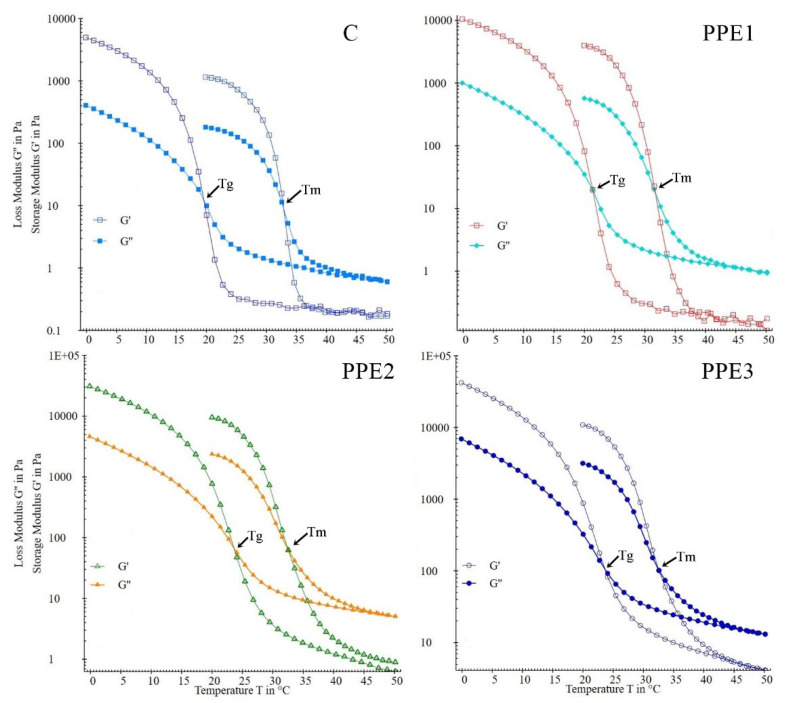
Effects of pomegranate peel extract (PPE) concentration on the storage (G′) and loss (G″) modulus of gelatin/sodium alginate composite solutions during heating and cooling process. (C: Control, without PPE; PPE1: gelatin/sodium alginate solution with 1% PPE; PPE2: gelatin/sodium alginate solution with 2% PPE; PPE3: gelatin/sodium alginate solution with 3% PPE; T_m_: Melting temperature (°C); T_g_: Gelation temperature (°C)).

The production of “Boba balls” occurs from two different processes, mainly the heating-swelling and cooling-molding of the polymer mix, and therefore, temperature ramp analysis of the “Boba” mix can provide significant information about the simulation of production. The storage ($G^{\prime }$) modulus and loss ($G^{\prime \prime }$) modulus from the temperature sweep test in LVR are illustrated in Figure 2. It is evident that the “Boba” mix was characterized as a viscoelastic structure due to higher $G^{\prime }$ than $G^{\prime \prime }$ up to 35 °C. In addition, $G^{\prime }$ and $G^{\prime \prime }$ values of the control were lower than in the PPE samples. The increasing modulus values could be attributed to the antioxidant and bioactive compounds of PPE, which have a positive influence on the final structural network formation of gelatin [16]. The heating and cooling cycles of the “Boba” mix gave significant information about the melting and gelation temperatures, respectively. During heating from 20 to 50 °C, the $G^{\prime }$ and $G^{\prime \prime }$ values of “Boba” mix decreased rapidly, and cross-over points were observed for samples, which were related to melting temperature (Table 1). The heating of the mix caused a single-strand arrangement of gelatin molecules and activated the molecular bonds, which resulted in the unfolding and full hydration of alginate, and hence, exhaustive dissolution [2]. $G^{\prime }$ and $G^{\prime \prime }$ values increased sharply as the temperature decreased, implying gel formation. The gelation process occurred through the transition from a single-strand arrangement to the triple-helix structure of gelatin, by means of hydrogen bonding, van der Waals forces, ionic interaction, hydrophobic association, and self-assembly [17]. The addition of PPE to the “Boba” mix slightly decreased the melting temperature (*p* > 0.05), whereas gelation temperature significantly increased (*p* < 0.05). The changes in gelation temperatures could be explained by the increased total soluble solid content of the “Boba” mix and the formation of triple-helix structures due to hydroxyl groups and hydrogen bonding. Additionally, the high molecular weight of PPE and SA provides a protective effect for the “Boba” mix gel structure [18]. Similar results were reported earlier by Liu, Xiao, Feng, Zhang, Li, Tu and Niu [2] for a konjac-glucomannan-SA “Boba” mix containing purple-sweet-potato-peel anthocyanin.

### 3.2. Encapsulation Efficiency and Bioactive Properties of “Boba Balls”

The encapsulation efficiency (EE), total phenolic content (TPC), antioxidant capacity, and total monomeric anthocyanin content (TMA) of PPE and PPE-enriched “Boba balls” are summarized in Table 1. The EE of “Boba balls” was determined based on TPC content, and therefore, the control sample displayed high EE results due to the TPC of pomegranate juice. The EE results of “Boba balls” decreased insignificantly with the PPE addition (*p* > 0.05). However, the TPC values of PPE were high (234.18 mg gallic acid equivalent/g of extract); the EE results of “Boba balls” may have decreased due to the high gelatin content of the “Boba” mix. The higher gelatin concentration resulted in the production of a porous, hydrophilic, and loose gel network structure, causing the encapsulated material to leach out during “Boba balls” production [19]. In a previous investigation, Liu, et al. [20] stated that the EE of alginate/gelatin hydrogels decreased when the gelatin concentration increased to 60 g/L. The authors explained this situation by the increasing viscosity of hydrogel, which caused the complex distribution of core material. In our study, the viscoelastic properties (Figure 2) of the “Boba” mix increased with the PPE addition. Therefore, the EE results decreased due to the inability to evenly distribute PPE within the polymer mix. Moreover, the gel structure of SA does not form immediately, and the contact with the Ca^+2^ ions is needed. Due to the hydrophilic nature of PPE, the encapsulation efficiency may have decreased due to the possibility that a small quantity of the extract was emitted into the CaCl_2_ solution [21].

The TPC value of PPE was determined to be 234.18 mg gallic acid equivalent/g of extract, which was comparable to the quantity reported in the literature [22]. The control sample produced with pomegranate juice displayed the lowest TPC value (25.50 mg gallic acid equivalent/g of extract), and the increasing PPE amount in “Boba balls” significantly increased the TPC values, and the highest result (*p* < 0.05) was observed from PPE3 (33.19 mg gallic acid equivalent/g of sample). CUPRAC, ABTS, and DPPH methods are the most widely used techniques for establishing the antioxidant capacity, and therefore these methods were used for PPE and “Boba balls”. Since the measurement principles of these methods are different from each other, the results obtained gave the expected results, but were not comparable with each other. As expected, the PPE was found to have the highest antioxidant capacity. Amongst the “Boba balls” samples, the control showed the lowest antioxidant capacity results due to the lower antioxidant capacity of pomegranate juice. The addition of PPE significantly improved the antioxidant capacity of “Boba balls” (*p <* 0.05). The increasing antioxidant capacity with PPE could be attributed to the secondary metabolites of pomegranate peels, such as phenolic compounds including flavonoids and tannins, which engage in metal chelating and scavenging activities [23].

The major pigments of pomegranate peel are dependent on the anthocyanin content, which belongs to the flavonoid family. The color change and bioactive compounds in the pomegranate peel are mainly affected by the variety, environmental conditions (temperature, rainfall, etc.), cultivation conditions, growing region, and ripening stage of the fruit to be harvested [24]. The TMA content of PPE was determined to be 80.54 mg cyaniding-3-glicoside eq/g, and our result was similar to the literature [25]. The lowest TMA value (34.63 mg cyaniding-3-glicoside eq/g) was determined for the control sample, and it was due to pomegranate juice. The addition of PPE to the “Boba” mix resulted in a significantly (*p* < 0.05) increased TMA value (*p* < 0.05).

### 3.3. Visual Appearance and Color of “Boba Balls”

**Figure 3:**
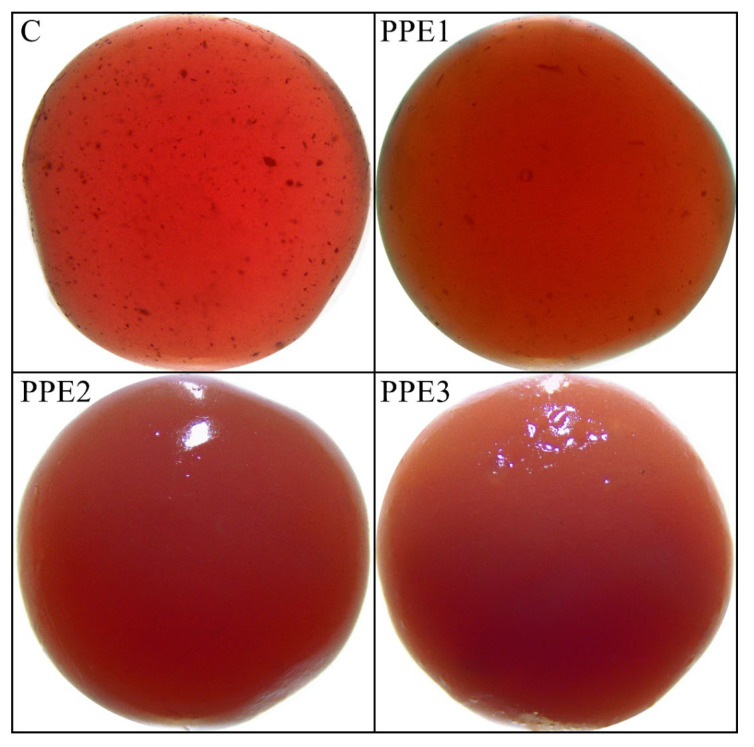
The visual appearances of “Boba balls” with stereomicroscope at 8× magnification. (C: Control, “Boba balls” without pomegranate peel extract (PPE); PPE1: “Boba balls” containing 1% PPE; PPE2: “Boba balls” containing 2% PPE; PPE3: “Boba balls” containing 3% PPE).

The visual appearance of “Boba balls” captured by a stereo microscope is shown in Figure 3, and the color results are summarized in Table 2. It is obvious in Figure 3 that “Boba balls” were successfully produced in spherical shapes. In addition, PPE addition to the “Boba” mix caused a cloudy appearance, and therefore, “Boba balls” not enriched with PPE were more transparent than PPE-containing samples. The control sample showed some particulate in the spherical “Boba” probably caused by the particulates in pomegranate juice, and the addition of PPE at 1% decreased the visibility of these particulates. Additionally, these particulates were not observed in PPE2 and PPE3 samples, possibly due to the increasing opacity. It can also be concluded that “Boba balls” had a homogeneous shape, surface structure, and stable size, meaning that the production of “Boba balls” was very stable. The color values of “Boba balls” revealed that PPE addition significantly (*p* < 0.05) affected the L* and b* values but did not affect a* values (*p >* 0.05). The lowest L* value (16.97) was observed for the PPE1 sample, while PPE3 showed the highest L* value (29.72). The significant change in the L* values could be attributed to the transparency and opacity of the samples, which is caused by light transmission through control and PPE1 samples, whereas PPE2 and PPE3 reflected the light and hence showed higher L* values. The b* values of the “Boba ball” samples were not significantly (*p* > 0.05) altered by the addition of PPE up to 3%. At 3% PPE, the “Boba balls” showed the highest b* results, which meant increased yellowness. However, the lower PPE concentrations caused a more greenish color of “Boba balls”. As expected, the typical color of “Boba balls” was reddish due to the dominant color of pomegranate juice, and the a* values were not significantly (*p* > 0.05) impacted by the PPE addition.

The total color differences (Δ*E*) of “Boba balls” were evaluated based on the control sample, which was produced without PPE addition. The results showed that there was no significant difference between PPE1 and PPE2 (*p* > 0.05), whereas PPE3 showed a significantly higher Δ*E* value than others (*p* < 0.05). The increasing PPE ratio caused higher Δ*E* values probably due to increasing L*, a* and b* values. According to the classification of Δ*E* values by Cserhalmi, et al. [26] “Boba balls” enriched with PPE displayed color differences between well-visible (3.0–6.0) and great (6.0–12.0).

### 3.4. Textural Properties

The hardness and elasticity of a hydrogel-type food product are crucial parameters that directly affect consumer acceptability. These parameters can easily and rapidly be determined by the texture profile analysis, and the results are provided in Table 2. Hardness, cohesiveness, gumminess and adhesiveness play an important part in swallowing physiology. Properties such as greater hardness, adhesiveness, and gumminess, and reduced cohesiveness are generally connected to a higher risk of aspiration [27]. The soft gel structure of “Boba balls” is widely utilized during the consumption of beverages such as milk tea, coffee, and yogurts. Therefore, a softer gel-forming agent is necessary to be easily chewed and swallowed. The hardness, gumminess, and chewiness values of samples decreased insignificantly (*p* > 0.05) for the control and PPE1 samples; however, the further addition of PPE significantly (*p* < 0.05) decreased the hardness. The decreasing hardness values after the 0.25% purple-sweet-potato-peel anthocyanin addition were also reported by Liu, Xiao, Feng, Zhang, Li, Tu and Niu [2]. The gel, which has a high springiness, is structurally divided into several large pieces upon the initial compression. In contrast, gels with lower springiness will structurally break into many small pieces at the end of the first compression [28]. Springiness values were stable for all samples up to 3% PPE addition and then significantly (*p* < 0.05) increased. The degree of difficulty required to break down the gel’s internal structure is an indication of the cohesiveness value. Although control, PPE1, and PPE2 showed the highest cohesiveness results, the lowest value was determined for PPE3. This means that the internal structure of “Boba balls” containing PPE at 1 and 2% was better than those containing 3% PPE. Resilience displays the degree to which a product strives to regain its initial height. PPE3 showed the lowest resilience and control, and PPE2 had the highest results.

### 3.5. FT-IR Spectrum

**Figure 4:**
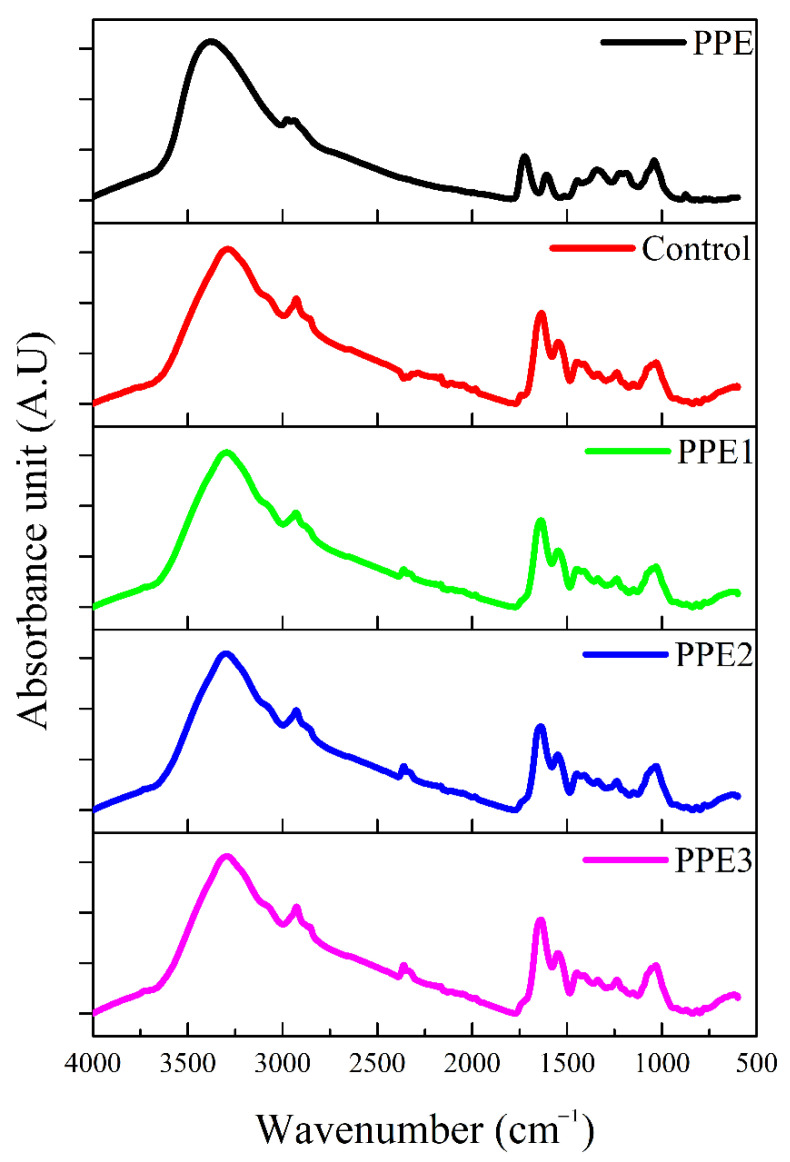
Effects of pomegranate peel extract (PPE) concentration on the Fourier transform infrared (FT-IR) spectrums. (PPE: pomegranate peel extract; C: Control without PPE; PPE1: “Boba balls” containing 1% PPE; PPE2: “Boba balls” containing 2% PPE; PPE3: “Boba balls” containing 3% PPE).

The potential molecular interactions between the “Boba” mix and core material (pomegranate peel extract) were investigated with the FT-IR spectrum. For this purpose, freeze-dried “Boba” mix without PPE, neat PPE, and “Boba balls” enriched with PPE at different ratios were analyzed, and the related images are illustrated in Figure 4. The FT-IR spectrum of PPE showed five major peaks located at the wavenumbers of 3377, 2972, 1725, 1594 and 1034 cm^−1^, which can be allocated to hydroxylic groups’ (alcohols, carboxylic acids, or phenols) stretching vibration bond; aliphatic C-H groups because of C-CH and C-CH_2_ bonds; COO groups of aldehyde and ketone, and carboxylic acid C=O groups; the C=O and C=C stretching vibration bonds; and the stretching bonding of CO and OH deformation in alcohols, respectively [29]. The “Boba” mix also showed the same peaks with the slight shifting of wavenumbers. The intensity of peaks located at 1635 and 1535 cm^−1^ for the “Boba” mix was higher than PPE because the “Boba” mix consists of gelatin, and these peaks (Amide I and II) are fingerprints for protein molecules. In addition, a similar backbone structure was observed for the “Boba balls” enriched with PPE. However, a new peak, which was not observed in the PPE and “Boba” mix, was detected for PPE-enriched “Boba balls” at the 2361 cm^−1^ wavenumber, and the intensity of this peak increased with increasing PPE ratio. This peak is highly related to carbonyl specific absorption [30]. Overall, the FT-IR spectra of PPE-enriched “Boba balls” revealed that PPE was successfully incorporated with “Boba” mix as a core material, and the observed shift in wavenumbers and new peak occurrence indicated a strong interaction between the PPE and “Boba balls”.

### 3.6. SEM Images

**Figure 5:**
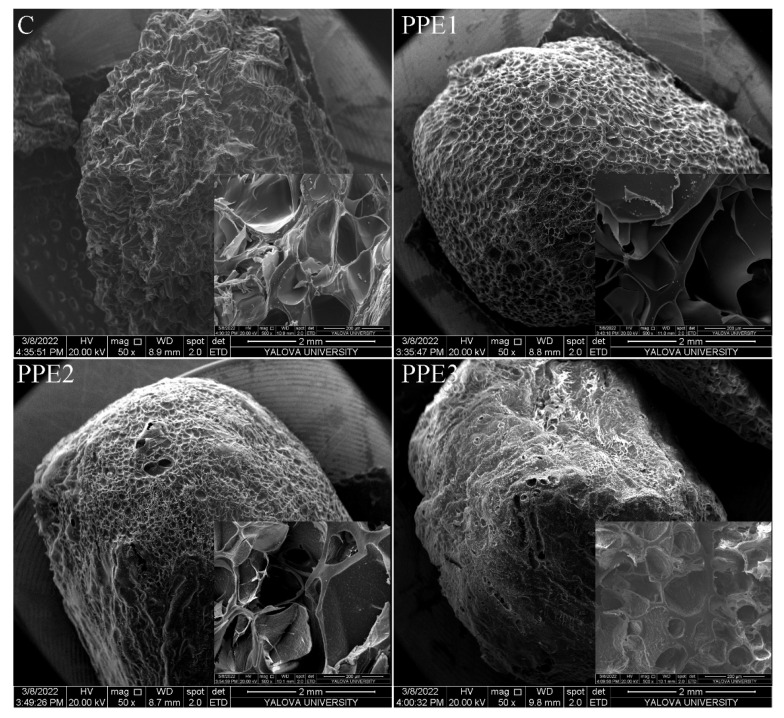
Scanning electron microscope (SEM) images of “Boba balls”. (C: Control, “Boba balls” without pomegranate peel extract (PPE); PPE1: “Boba balls” containing 1% PPE; PPE2: “Boba balls” containing 2% PPE; PPE3: “Boba balls” containing 3% PPE).

The interactions between polymers used for “Boba ball” production can be further explained by morphological characterization, and for this purpose, the freeze-dried “Boba balls” were investigated with an electron microscope. The images were captured from the surface (50×) and cross-section (500×) of broken “Boba balls” by liquid nitrogen (Figure 5). The deformity and heterogeneous surface structure with a wrinkled shape of control sample could be related with the high sublimation rates of ice crystals during freeze-drying [2]. However, the surface structure was significantly changed by the addition of PPE, and smoother surfaces occurred as the PPE concentration increased. The increasing PPE concentration caused a denser surface structure with smaller voids, and more junction zones were formed, probably due to the cross-linking of PPE-gelatin and SA [2]. In addition, the smoother surfaces of PE-added “Boba balls” may be associated with the high sugar content of PPE. In general, uniform spherical microbeads are more attractive than non-uniform shapes for drug delivery and controlled-release applications because uniform microbeads offer the prolonged and precise release of encapsulated compounds [31]. The cross-sectional images of the samples clearly revealed that the increasing PPE concentration increased the connections between the polymers, and PPE3 showed a denser cross-section without huge voids. Furthermore, it was confirmed that the cross-sectional morphology of “Boba balls” at 2 and 3% PPE showed a more uniform and compact network arrangement with less pores in the gels. The dense internal structure of “Boba balls” could also improve the controlled release of PPE. These results can also be well corroborated with the uniform spherical shapes of PPE2 and PPE3, as well as encapsulation efficiency, rheology and texture analysis.

### 3.7. In Vitro Digestion of “Boba Balls”

**Figure 6:**
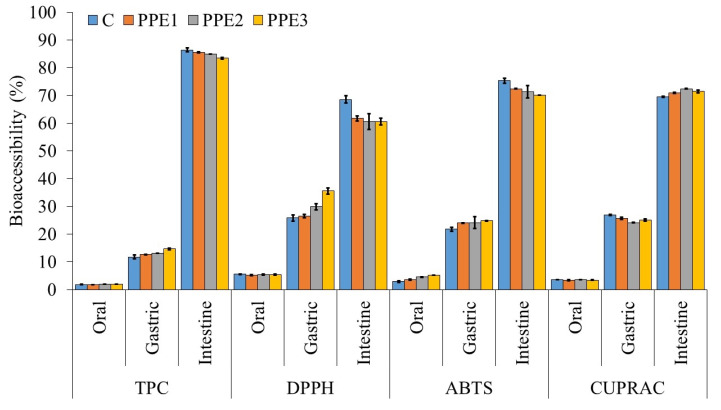
The in vitro bio-accessibility of “Boba balls” containing pomegranate peel extract (PPE) at various concentrations. (C: Control without PPE; PPE1: “Boba balls” containing 1% PPE; PPE2: “Boba balls” containing 2% PPE; PPE3: “Boba balls” containing 3% PPE).

The in vitro digestibility of coated and/or encapsulated bioactive substances is very important because most of the bioactive substances are required to pass through the mouth and stomach without being damaged and to be released in the intestinal medium, thus increasing the bio-availability. In vitro bio-accessibility of PPE-loaded “Boba balls” was evaluated by the assessment of TPC and antioxidant capacity. The findings are depicted in Figure 6, and it can clearly be seen that the bio-accessibility of phenolic and antioxidant compounds in “Boba balls” were lower than 10% in an oral medium, which meant that these bioactive compounds were successfully protected against oral digestive fluids. In addition, the initial bio-accessibility of bioactive compounds in an oral medium may possibly be associated with the phenolic compounds on the surface of “Boba balls”. The TPC bio-accessibility in the gastric medium ranged from 11.75 to 14.66% and increased significantly by the increasing of PPE ratio. The antioxidant capacity tests in the gastric condition were higher than the TPC results, and they were also affected by the PPE concentration. The increasing bio-accessibility in the gastric medium was mainly caused by the variation in ionic composition, pH, and gastric enzymes, which caused the leakage of phenolic compounds [32]. Moreover, during the 2 h duration of gastric conditions, “Boba balls” showed TPC bio-accessibility lower than 15%, and antioxidant capacity lower than 40 and 30% for DPPH, ABTS, and CUPRAC, respectively. This situation clearly revealed that the strong biopolymer matrix network restrained the seepage of bioactive compounds from “Boba balls” to the stomach. Less discharge in the stomach is advantageous since more bioactive compounds would be absorbed in the intestinal medium. The bio-accessibility of bioactive compounds in “Boba balls” in the intestinal medium showed the highest values, probably due to the intense electronic repulsion between alginate molecules, which triggered the degradation of the hydrogel [33]. In a previous study, uncoated PPE showed only 22.46% bio-accessibility in the intestinal medium, and 87.83% of the initial phenolic compounds in PPE were degraded in the oral phase, whereas 46.60% of TPC was degraded in gastric conditions [34]. It can be clearly concluded from our results that the production of “Boba balls” enriched with PPE significantly improves the bio-accessibility of bioactive compounds in gastrointestinal digestion.

### 3.8. In Vitro Release Kinetic of “Boba Balls”

**Figure 7:**
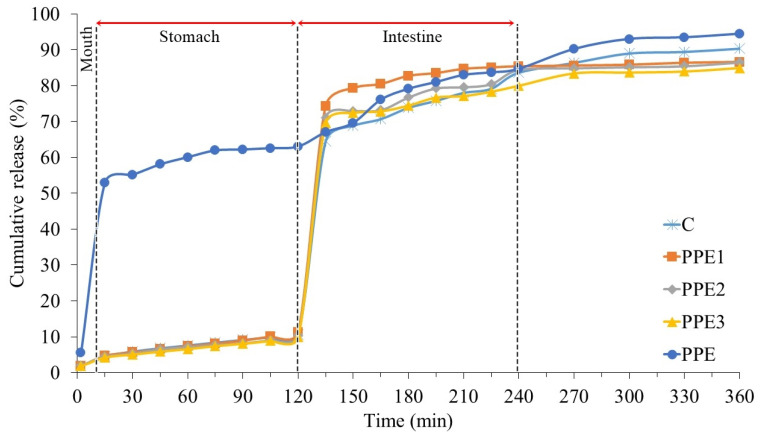
Release pattern of pomegranate peel extract (PPE) from “Boba balls” in the simulated gastrointestinal conditions. (PPE: pomegranate peel extract; C: Control without PPE; PPE1: “Boba balls” containing 1% PPE; PPE2: “Boba balls” containing 2% PPE; PPE3: “Boba balls” containing 3% PPE).

The in vitro release kinetic of phenolic compounds in the “Boba balls” in the stomach and intestinal environments was evaluated with widely used release models. The cumulative percentage release vs. time graph of TPC in PPE and “Boba balls” is described in Figure 7, and the related model parameters are provided in Table 3. The highest release percentage for oral and gastric conditions was determined from the PPE, which showed that the phenolic compounds of PPE should be protected via encapsulation for better biological activity. However, TPC release from PPE in the intestinal medium was lower than “Boba balls” at the beginning of the digestion in the intestine, and in contrast to “Boba balls”, attained a maximum level at the conclusion of digestion. The release rate of TPC in the oral medium was very low, whereas in the gastric medium, the release rate linearly increased during the 2 h duration. The increasing rate could be attributed to phenolic compounds attached to the surface of “Boba balls”. However, the release rate reached the highest values when samples passed through the intestinal medium, probably due to the effectiveness of bile salt, which increased the leakage of active compounds, and the pH value of the intestine medium, which resulted in the solubility of alginate [35]. The release data were fitted to three different models, including Korsmeyer–Peppas, Peppas–Sahlin, and Makoid–Banakar, the most used models for drug-release studies. As seen in Table 3, all models, except for Korsmeyer–Peppas in the intestinal medium, showed high R^2^ values (>0.950) both for the stomach and intestine medium. In the Korsmeyer–Peppas model, the exponent *n* describes the release mechanism and highly depends on the geometry of the micro particles. If *n =* 0.43, spherical-shaped micro particles show diffusion-controlled release or Fickian diffusion, whereas if *n* = 0.85, micro particles indicate swelling-controlled release or case II transport of active compounds. However, in some cases in which *n* values are between 0.43 and 0.85, the release behavior of micro particles can be defined as the superposition of both phenomena, called anomalous transport [36]. In our study, “Boba balls” showed *n* values lower than 0.43 both for gastric and intestinal conditions and showed a quasi-Fickian diffusion of TPC [37]. These results reveal that the release mechanism of PPE from “Boba balls” was diffusion-controlled, which means the concentration gradient was the main trigger for release [38,39].

### 3.9. Sensory Properties

**Figure 8:**
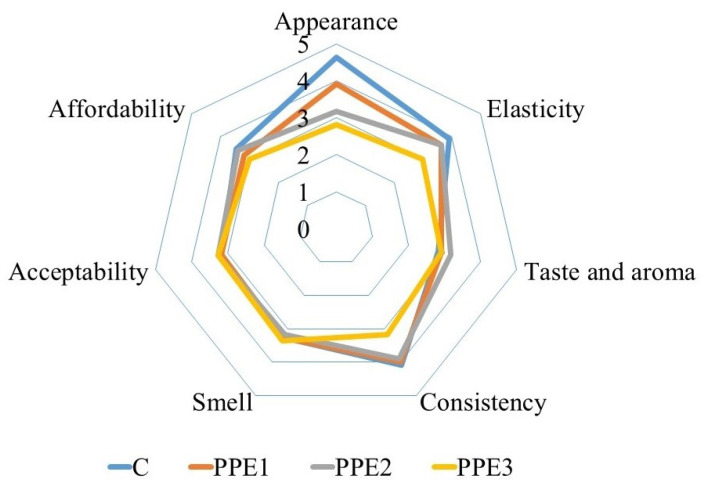
Sensory evaluation of “Boba balls” produced with gelatin/sodium alginate composite containing pomegranate peel extract (PPE) at various concentrations. (C: Control without PPE; PPE1: “Boba balls” containing 1% PPE; PPE2: “Boba balls” containing 2% PPE; PPE3: “Boba balls” containing 3% PPE).

The sensory properties of “Boba balls” enriched with PPE at various concentrations are depicted in Figure 8. The addition of PPE to “Boba balls” had insignificant (*p < 0.05*) effects on some sensory properties of the final product but did not change other sensory properties such as taste and aroma, smell, acceptability, and affordability. However, the appearance, elasticity, and consistency scores of “Boba balls” decreased with an increasing PPE ratio. The changes in sensory scores are well supported by Figure 3, showing that the opacity of “Boba balls” increased when the PPE ratio increased; hence, the appearance scores decreased. Moreover, the increasing PPE ratio caused the decreasing elasticity. The decreased elasticity may be due to increasing the total dry matter of “Boba balls”; this result is well supported by Figure 2, in which the elastic modulus increased with increasing PPE ratio. When the consistency values from the panelists were examined, it was clear that all samples, except for PPE3, had similar consistency, and the lowest consistency result was observed from PPE3. As a general evaluation of sensory scores, the PPE enrichment of “Boba balls” had no significant effect on the acceptability and affordability of the final products, which were close to the control sample. Thus, PPE could be used as supplementary material for “Boba balls” to enhance antioxidant and bio-accessibility properties.

## 4. Conclusions

In this study, “Boba balls” were successfully enriched with pomegranate peel extract (PPE), and then the bioactivity, bio-accessibility, release kinetic, physical, chemical, and morphological properties, as well as sensory properties, of “Boba balls” were characterized. PPE addition significantly increased the gelation temperature, whereas no significant (*p* > 0.05) effect was observed on the encapsulation efficiency and melting temperature. The highest bioactive properties, such as antioxidant capacity, total monomeric anthocyanin, and total phenolic content, were observed for PPE, and as expected, increasing PPE concentration in “Boba balls” significantly (*p* < 0.05) improved these results. The visual appearance of “Boba balls” changed and became more opaque as PPE concentration increased. The hardness and chewiness of “Boba balls” significantly decreased with PPE addition. FT-IR results clearly revealed the chemical bonds occurring between PPE and “Boba balls”. Additionally, more junction zones were observed from the microstructural images of PPE-incorporated samples. The in vitro bio-accessibility of “Boba balls” clearly revealed that PPE was successfully covered by the “Boba” mix and increased the gastrointestinal bio-accessibility. Moreover, the release kinetic of PPE from “Boba balls” was modeled by widely used drug-release kinetic models with a higher *R^2^* than 0.85. Sensory scores clearly showed that the smell, taste, aroma, overall acceptability, and affordability of “Boba balls” did not change with the PPE addition. At the same time, appearance, elasticity, and consistency decreased. Considering all these results, PPE can be successfully encapsulated in the “Boba balls”. It can also be a valuable supplementary material for improving not only the bioactivity of “Boba balls” but also the bio-accessibility.

## Figures and Tables

**Table 1:** The melting and gelation temperatures of “Boba” mix and bioactive properties of “Boba balls”.

Properties	PPE	C	PPE1	PPE2	PPE3
T_m_	-	32.88 ± 1.75	31.70 ± 0.98	32.52 ± 1.57	32.53 ± 1.18
T_g_	-	19.56 ± 0.32 ^c^	21.38 ± 0.45 ^b^	23.60 ± 0.57 ^a^	23.65 ± 0.86 ^a^
EE	-	75.25 ± 9.21	72.74 ± 3.99	71.59 ± 2.72	68.52 ± 0.51
TPC	234.18 ± 3.61	25.50 ± 0.01 ^d^	28.48 ± 0.51 ^c^	30.86 ± 0.02 ^b^	33.19 ± 0.27 ^a^
ABTS	1465.46 ± 26.71	7.96 ± 0.32 ^d^	11.69 ± 0.99 ^c^	27.56 ± 0.01 ^b^	50.49 ± 1.49 ^a^
CUPRAC	3215.66 ± 48.69	7.45 ± 0.25 ^d^	16.43 ± 0.76 ^c^	22.52 ± 0.67 ^b^	36.52 ± 0.58 ^a^
DPPH	458.74 ± 33.25	8.15 ± 0.31 ^d^	18.66 ± 0.15 ^c^	32.75 ± 1.09 ^b^	35.54 ± 1.13 ^a^
TMA	80.54 ± 0.06	34.63 ± 0.04 ^d^	41.39 ± 0.15 ^c^	49.10 ± 0.04 ^b^	54.35 ± 0.17 ^a^

Values are means ± standard deviation. ^a–d^ Means within the same row with different letters are significantly different (*p* < 0.05). T_m_: Melting temperature (°C); T_g_: Gelation temperature (°C); EE: Encapsulation efficiency (%); TPC: Total phenolic content (mg gallic acid equivalent/g of extract); ABTS, CUPRAC and DPPH are antioxidant capacity measurement methods as µmole trolox/g; TMA: Total monomeric anthocyanin content (mg cyaniding-3-glucoside equivalents/g); C: “Boba balls” without PPE addition, Control; PPE: Pomegranate peel extract; PPE1: “Boba balls” enriched with 1% PPE; PPE2: “Boba balls” enriched with 2% PPE; PPE3: “Boba balls” enriched with 3% PPE.

**Table 2:** Color and textural properties of “Boba balls”.

	C	PPE1	PPE2	PPE3
L*	20.96 ± 0.37 ^c^	16.97 ± 0.49 ^d^	26.24 ± 3.10 ^b^	29.72 ± 1.78 ^a^
a*	28.39 ± 1.88	26.39 ± 0.44	31.48 ± 2.51	28.47 ± 1.94
b*	12.25 ± 0.19 ^b^	10.51 ± 0.31 ^b^	12.48 ± 1.37 ^b^	18.04 ± 1.89 ^a^
Δ*E*	-	4.60 ± 0.27 ^b^	5.02 ± 0.26 ^b^	6.97 ± 0.98 ^a^
Hardness (g)	424.18 ± 95.13 ^a^	398.76 ± 50.66 ^a^	275.88 ± 48.22 ^b^	116.53 ± 25.74 ^c^
Springiness	0.98 ± 0.01 ^b^	0.98 ± 0.01 ^b^	0.98 ± 0.01 ^b^	1.03 ± 0.04 ^a^
Cohesiveness	0.80 ± 0.01 ^a^	0.82 ± 0.01 ^a^	0.83 ± 0.02 ^a^	0.50 ± 0.09 ^b^
Gumminess (g)	340.22 ± 79.42 ^a^	325.2 ± 42.06 ^a^	228.33 ± 42.62 ^b^	60.41 ± 21.52 ^c^
Chewiness (g)	334.17 ± 77.91 ^a^	319.15 ± 41.1 ^a^	224.36 ± 41.46 ^b^	61.37 ± 20.38 ^c^
Resilience	0.88 ± 0.02 ^a^	0.84 ± 0.02 ^bc^	0.87 ± 0.03 ^ab^	0.81 ± 0.06 ^c^

Values are means ± standard deviation. ^a–d^ Means within the same row with different letters are significantly different (*p* < 0.05). L*: Lightness; a*: redness; b*: yellowness; Δ*E*: Total color difference; C: “Boba balls” without PPE addition, Control; PPE1: “Boba balls” enriched with 1% PPE; PPE2: “Boba balls” enriched with 2% PPE; PPE3: “Boba balls” enriched with 3% PPE.

**Table 3:** The release model parameters of total phenolic compounds from “Boba balls” hydrogel to the stomach and intestinal environments.

Medium	Model	Coefficients	C	PPE1	PPE2	PPE3
Gastric conditions	Korsmeyer–Peppas	k	1.885	1.402	1.400	1.490
		n	0.351	0.422	0.410	0.378
		R^2^	0.9797	0.9647	0.9609	0.9742
	Peppas–Sahlin	k_1_	1.451	1.248	1.289	1.289
		k_2_	0.657	0.427	0.371	0.426
		m	0.227	0.274	0.274	0.252
		R^2^	0.9821	0.9677	0.9656	0.9782
	Makoid–Banakar	K_MB_	1.677	1.324	1.868	1.747
		n	0.385	0.439	0.328	0.332
		c	0.000	0.000	−0.001	0.000
		R^2^	0.9801	0.9648	0.9625	0.9748
Intestinal conditions	Korsmeyer–Peppas	k	34.639	43.597	39.809	39.718
		n	0.182	0.141	0.153	0.148
		R^2^	0.9547	0.8411	0.8983	0.9036
	Peppas–Sahlin	k_1_	33.307	40.527	38.244	39.552
		k_2_	−3.048	−4.706	−4.302	−4.712
		m	0.285	0.301	0.286	0.271
		R^2^	0.9798	0.9738	0.9701	0.9688
	Makoid–Banakar	K_MB_	30.543	35.333	33.768	34.640
		n	0.232	0.239	0.226	0.211
		c	0.001	0.002	0.001	0.001
		R^2^	0.9722	0.9569	0.9563	0.9534

## Data Availability

The data presented in this study are available on request from the corresponding author. The data are not publicly available due to privacy.
